# Do Variants in the *GST* Detoxification Genes Affect the Risk of Lung Cancer?

**DOI:** 10.1371/journal.pmed.0030174

**Published:** 2006-03-07

By the year 2020, global deaths from noncommunicable diseases are expected to increase by 77%. The projected rise is mainly due to the aging of the population and to an increase in the numbers of people exposed to tobacco. While antismoking campaigns have met with some success in developed countries, the tobacco epidemic is growing in many of the world's developing and most populous countries. Tobacco (a major risk factor not only for lung cancer but also for chronic obstructive pulmonary diseases such as emphysema) is expected to kill more people than any single disease, surpassing even the HIV epidemic.

The link between tobacco smoke and lung cancer is striking, but not all smokers get lung cancer, and not all lung cancer patients have a history of first- or second-hand exposure to tobacco smoke. Tobacco smoke contains carcinogens such as benzopyrene, which can cause mutations in the DNA of cells it comes in contact with, such as lung epithelial cells. Cytosolic enzymes such as those of the glutathione S-transferase (GST) family are part of the human body's armor against environmental carcinogens; they catalyze the detoxification of reactive electrophilic compounds like benzopyrene. Like all human genes, those encoding members of the GST family exist in multiple variant forms or alleles. Some of these alleles encode less-active or completely inactive versions of the detoxifying enzymes, and, therefore, might convey increased risk for the development of cancers with strong environmental determinants, and lung cancer in particular.

Although more than 100 studies have tested the hypotheses that particular *GST* alleles either predispose to or protect against lung cancer, the results have been inconsistent, with some studies reporting strong associations and others failing to replicate these findings. Individual association studies are notoriously prone to error, and attempts to combine results from several studies have been complicated by the fact that allele frequencies for many of the alleles differ between populations, by differences in smoking habits, and by differences in criteria for the selection of suitable control groups.

To summarize the current evidence comprehensively and attempt to resolve the controversy, Zheng Ye and colleagues undertook a large meta-analysis of 130 published studies that had examined associations between one or several of five *GST* alleles and lung cancer. The five alleles included the *GSTM1* and *GSTT1* null alleles, two missense alleles in *GSTP1*, and one intron polymorphism in *GSTM3*. (The null alleles abolish enzyme activity; the three other alleles encode enzymes with reduced activity.) A total number of 23,452 lung cancer cases and 30,397 controls were included in the meta-analysis. Ye and colleagues corresponded with the individual research groups to obtain the data in tabular form and—for a few of the studies—additional data that were not included in the original publications.

When taking all the existing evidence together, neither the I105V or A114V polymorphisms in *GSTP1* nor the *GSTM3* intron 6 polymorphism were found to be associated with increased risk of lung cancer. The two “null” polymorphisms in GSTM1 and *GSTT1* showed a weak association. However, as Ye and colleagues discuss, it is possible that the weak overall link results from bias toward publication of positive results, especially for smaller studies. This is consistent with the result that the researchers obtained when they restricted their analysis to larger studies: comparing only cases and controls from studies that had at least 500 participants resulted in no significant associations between any of the alleles and lung cancer.

While meta-analyses such as this one have their own limitations, the results reported here make it unlikely that any of the five polymorphisms convey a substantially enhanced lung cancer risk in the general population. This does not exclude the possibility that other alleles in the four genes examined here, alleles in other *GST* genes, or combinations of *GST* alleles do exert substantial influences on an individual's lung cancer risk. As the authors conclude, the chances for discovering such links in future studies are likely much higher if such studies are large and carefully designed.

